# INTERFACE OF INFORMAL AND FORMAL SUPPORT IN THE LINKAGE BETWEEN COGNITIVE DECLINE AND LONELINESS

**DOI:** 10.1093/geroni/igac059.2633

**Published:** 2022-12-20

**Authors:** Soobin Park, Sojung Park, Takashi Amano, BoRin Kim

**Affiliations:** Washington University in St. Louis, St. Louis, Missouri, United States; Washington University in St. Louis, St. Louis, Missouri, United States; Rutgers University-Newark, Newark, New Jersey, United States; University of New Hampshire, Durham, New Hampshire, United States

## Abstract

Older adults with cognitive impairment are at risk of higher loneliness due to increasing challenges to retain essential social skills and decreasing social networks. This study explores ways to reduce the relationship between cognitive function and loneliness. Drawing on the cognitive discrepancy theory of loneliness, we hypothesized loneliness may be reduced by improving an individual’s actual level of social interaction, creating opportunities for social relationships. This study examined complex relationships among informal (frequency of social contact with family members and friends) and formal support (use of home- and community-bound services, HCBS), and loneliness, among older adults with different levels of cognitive impairment. Data came from the Health and Retirement Study (2012) (N=651) with the sample included respondents 51+ years who completed a modified Telephone Interview for Cognitive Status. Results from hierarchical regression showed older people with a lower level of cognitive function were less likely to experience loneliness (b=-.15, p<.01). Although frequent social contact with family and friends reduces loneliness (b=-.24, p<.001), it did not moderate the relationship between cognitive function and loneliness. Interestingly, the use of HSBC also turned out to be not a significant protective factor. Our findings suggest merely increasing social interaction levels with family and friends may not necessarily curb loneliness for older adults with cognitive impairment. Given the current characteristics and nature of HCBS primarily focus on providing basic physical needs of older adults, prioritizing the development of emotional comfort and recreational activities that alleviate their psychological loneliness is essential, especially amidst the pandemic.

